# Supplementary material for: Diffusion tensor imaging in Chediak Higashi Disease
Source: medRxiv. 2025 Aug 10:2025.08.06.25332949. Preprint. [Version 1] doi: 10.1101/2025.08.06.25332949 (PMC12443054; doi:10.1101/2025.08.06.25332949)
Supplement: 1 [file NIHPP2025.08.06.25332949V1-supplement-1.pdf]

medRxiv preprint doi: https://doi.org/10.1101/2025.08.06.25332949; this version posted August 10, 2025. The copyright holder for this preprint (which was not certified by peer review) is the author/funder, who has granted medRxiv a license to display the preprint in perpetuity. This article is a US Government work. It is not subject to copyright under 17 USC 105 and is also made available for use under a CC0 license.

Diffusion tensor imaging in Chediak Higashi Disease

Supplementary Material

Table of Contents

Methods……………………………………………………………………………….…*19*

Supplement A: Neurotypical Controls Participants ……..………………………………………*20*

Supplement B: DTI Acquisition ……..………*21*

Supplement C: Linear Mixed Effects Modeling…………………………………………………*23*

Results…………………………………………………………………………………...*24*

Supplement D: Diffusion Tensor Imaging Analysis Supplementary Data ………………………*24*

Supplement E: DTI LMEM Estimates, Standard Errors, and Graphs…………………………….*30*

Supplement F: DTI LMEM Graphs…………………………...……………………………….*34*

Supplement G: Correlational Fiber Tractography Supplementary Data…………………………*.36*

Supplement H: Correlational Fiber Tractography Localization…..……………………………...*38*

Supplementary References……………………………………………………………...…*40*

medRxiv preprint doi: https://doi.org/10.1101/2025.08.06.25332949; this version posted August 10, 2025. The copyright holder for this preprint (which was not certified by peer review) is the author/funder, who has granted medRxiv a license to display the preprint in perpetuity. This article is a US Government work. It is not subject to copyright under 17 USC 105 and is also made available for use under a CC0 license.

Supplementary Methods

Supplement A: Neurotypical Control Participant Characteristics

Neurotypical controls (NC) were age- and sex-matched to CHD participants (Figure B1). Four NC participants were selected for each DTI scan. 100 participants were included for the DTI analysis. Participants were selected from the following datasets:

Calgary Neurotypical Controls[2, 3]
16 participants (8 females) were included in the DTI analysis from the "Calgary Preschool magnetic resonance imaging (MRI)" dataset.

NIMH[4,5]
35 participants (12 females) were in included in the DTI analysis from "The National Institute of Mental Health (NIMH) Intramural Healthy Volunteer Dataset"

UCLA[6,7]
12 participants (12 males) from the "UCLA Consortium for Neuropsychiatric Phenomics LA5c Study" were included in the DTI analysis.

AOMIC-PIOP1[8,9]
20 participants (8 females) from the "Amsterdam Open MRI Collection (AOMIC) Population Imaging of Psychology (PIOP)" dataset were included in the DTI analysis.

AOMIC[8,10]
17 participants (17 males) from the "Amsterdam Open MRI Collection (AOMIC)" dataset were included in the DTI analysis.

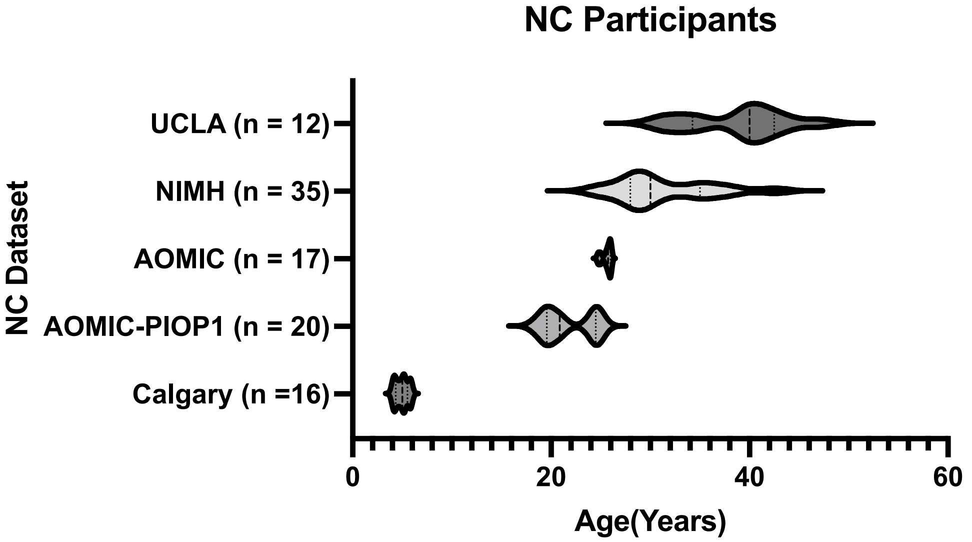

Figure A1. NC participant age. Each NC dataset is on a separate row for each of the five datasets used in this study.

medRxiv preprint doi: https://doi.org/10.1101/2025.08.06.25332949; this version posted August 10, 2025. The copyright holder for this preprint (which was not certified by peer review) is the author/funder, who has granted medRxiv a license to display the preprint in perpetuity. This article is a US Government work. It is not subject to copyright under 17 USC 105 and is also made available for use under a CC0 license.

Supplement B: DTI Acquisition

Chediak-Higashi Disease Participants DTI Acquisition

DTI scans for CHD participants were conducted on a 3T Phillips (Philips Healthcare, Best, The Netherlands) Achieva MR System with an 8-channel SENSE head coil. DTI scans were collected with the following parameters: TR/TE = 6400/100 ms, 15-gradient encoding directions, b-values = 0 and 1000 s/mm^2, voxel size = 1.875mm × 1.875mm × 2.5mm, slice thickness = 2.5 mm, acquisition matrix = 128 × 128, NEX = 1, FOV = 24 cm.

Calgary Neurotypical Controls[2, 3]

A General Electric 3T MR750w system with a 32-channel head coil was used for scanning all Calgary neurotypical controls using a single shot spin echo-planar imaging sequence. DTI images were acquired with the following parameters for Calgary neurotypical controls: TR/TE=6750/79 ms, 30-gradient encoding directions, b-values = 0 and 750 s/mm^2, voxel size = 1.6mm×1.6mm×2.2mm, slice thickness = 2.2 mm, FOV = 20 cm.

NIMH[4,5]

A 3T General Electric 3T Discovery MR750w system with a 32-channel head coil was used for scanning all NIMH neurotypical controls using a single shot spin echo-planar imaging sequence. DTI images were acquired with the following parameters for NIMH neurotypical controls: TR/TE=7800/61 ms, 24-gradient encoding directions, b-values = 0 and 1000 s/mm^2, voxel size = 2mm×2mm×2mm, slice thickness = 2.0 mm, FOV = 23.2 cm as modified from the Alzheimer's Disease Neuroimaging Initiative (ADNI3).

UCLA[6,7]

A Siemens 3T Trio scanner was used to acquire this data. DTI using an echo-planar sequence with the following parameters for UCLA neurotypical controls: TR/TE = 9000/93 ms, 64-gradient encoding directions, b-values = 0 and 1000 s/mm^2, voxel size = 2mm×2mm×2mm, slice thickness = 2.0 mm.

medRxiv preprint doi: https://doi.org/10.1101/2025.08.06.25332949; this version posted August 10, 2025. The copyright holder for this preprint (which was not certified by peer review) is the author/funder, who has granted medRxiv a license to display the preprint in perpetuity. This article is a US Government work. It is not subject to copyright under 17 USC 105 and is also made available for use under a CC0 license.

AOMIC-PIOP1[8,9]

A Philips 3T Achieva system with a 32-channel head coil. DTI images were acquired using single shell spin-echo diffusion-weighted imaging with the following parameters for AOMIC-PIOP1 neurotypical controls: TR/TE = 7387/86 ms, 32-gradient encoding directions, b-values = 0 and 1000 s/mm^2, flip angle = 90°, FOV = 22.4 × 22.4 × 12.0 cm, voxel size= 2x2x2 mm, and 60 slices.

AOMIC[8,10]

MRI data were acquired using a Philips 3T Intera scanner with a 32-channel head coil. DTI images were acquired using single shell spin-echo diffusion-weighted imaging with the following parameters for AOMIC neurotypical controls: TR/TE = 6312/74 ms, 32-gradient encoding directions b-values = 0 and 1000 s/mm^2, flip angle = 90°, FOV = 22.4 × 22.4 × 12.0 cm, voxel size= 2x2x2 mm, and 60 slices.

medRxiv preprint doi: https://doi.org/10.1101/2025.08.06.25332949; this version posted August 10, 2025. The copyright holder for this preprint (which was not certified by peer review) is the author/funder, who has granted medRxiv a license to display the preprint in perpetuity. This article is a US Government work. It is not subject to copyright under 17 USC 105 and is also made available for use under a CC0 license.

Supplement C: Linear Mixed Effects Modeling

Linear mixed effects modeling was built in R with the LME4 package. The interaction between the cohort and biological age was tested using the sample code below:

Model 1:

```
model1 <- lmer(DTI Metric ~ Age * CHD + Sex + (1|Participant), dataset)
summary(lmer)
```

Model 2:

```
model2 <- lmer(DTI Metric ~ Age + CHD + Sex + (1|Participant), dataset)
summary(lmer)
```

Likelihood-ratio test:

```
fm.anova <- anova(model1,model2)
summary(fm.anova)
```

Predicted Brain Age – Corresponds to the predicted brain age determined by BrainStructuresAges for the structure being analyzed
Cohort – Corresponds to the comparison between cohorts as presented in Table 1.
Sex – Corresponds to the participants' biological sex
Age – Corresponds to the participants' chronological age
Participant – Each participant was given a distinct number to account for repeated measures (subject level random intercept).

medRxiv preprint doi: https://doi.org/10.1101/2025.08.06.25332949; this version posted August 10, 2025. The copyright holder for this preprint (which was not certified by peer review) is the author/funder, who has granted medRxiv a license to display the preprint in perpetuity.
This article is a US Government work. It is not subject to copyright under 17 USC 105 and is also made available for use under a CC0 license.

Supplementary Results

Supplement D: Diffusion Tensor Imaging Analysis Supplementary Data

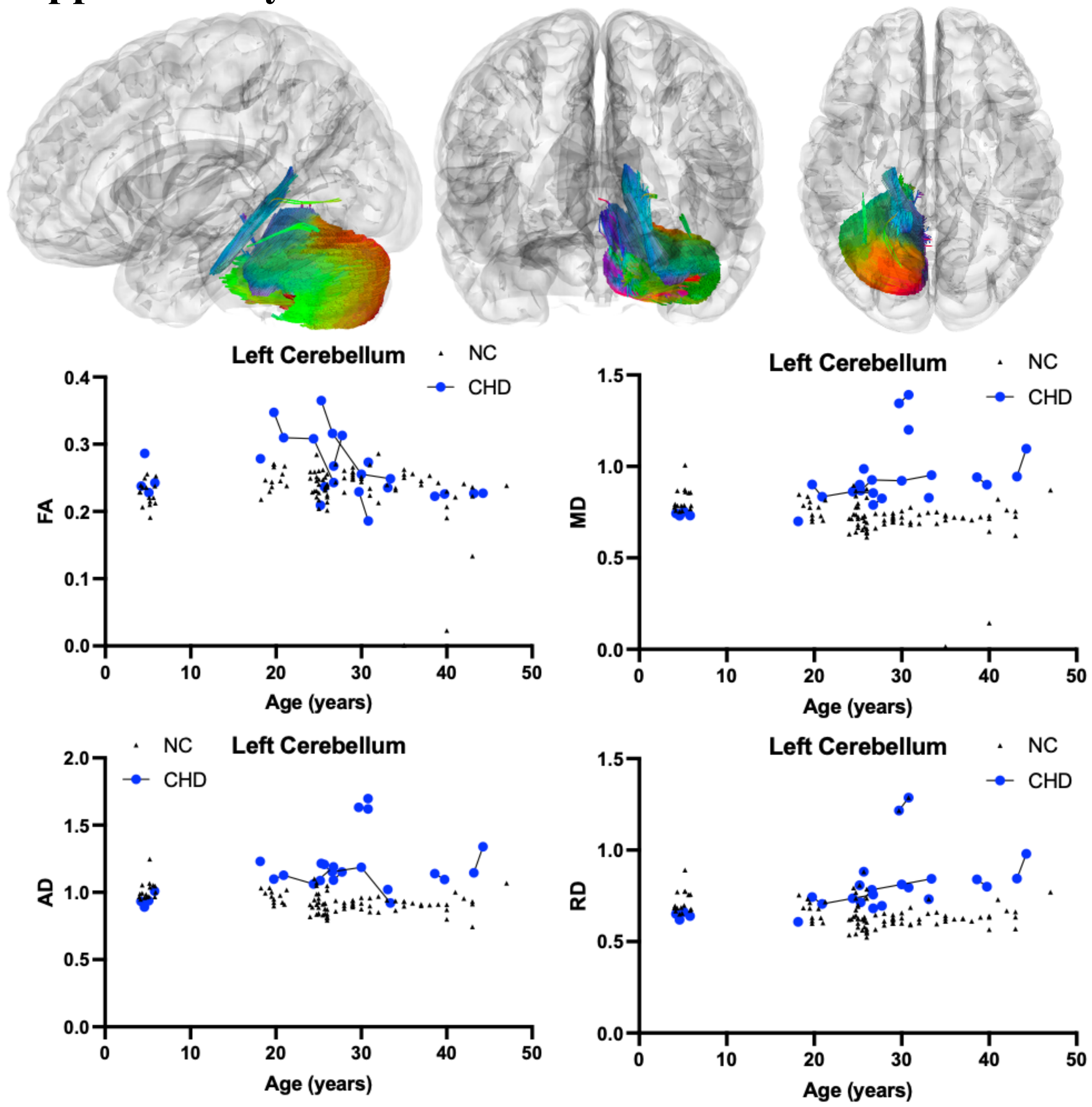

Figure D1. Atlas Based Fiber Tractography of the left cerebellum demonstrating age related effects on A.) fractional anisotropy (FA), B.) mean diffusivity (MD), C.) radial diffusivity (RD), D.) axial diffusivity (AD) between CHD participants (blue) and NC controls (black triangles). There was no statistically significant interaction between participant age and the presence of CHD on left cerebellum FA ($\chi^2(1) = 0.47$, p-value$_{corrected} = 1.000$). There was a statistically significant positive interaction between participant age and the presence of CHD on left cerebellar MD ($\chi^2(1) = 21.73$, p-value$_{corrected} = 0.0001$), AD ($\chi^2(1) = 15.51$, p-value$_{corrected} = 0.0030$), and RD ($\chi^2(1) = 27.04$, p-value$_{corrected} < 0.0001$).

medRxiv preprint doi: https://doi.org/10.1101/2025.08.06.25332949; this version posted August 10, 2025. The copyright holder for this preprint (which was not certified by peer review) is the author/funder, who has granted medRxiv a license to display the preprint in perpetuity.
This article is a US Government work. It is not subject to copyright under 17 USC 105 and is also made available for use under a CC0 license.

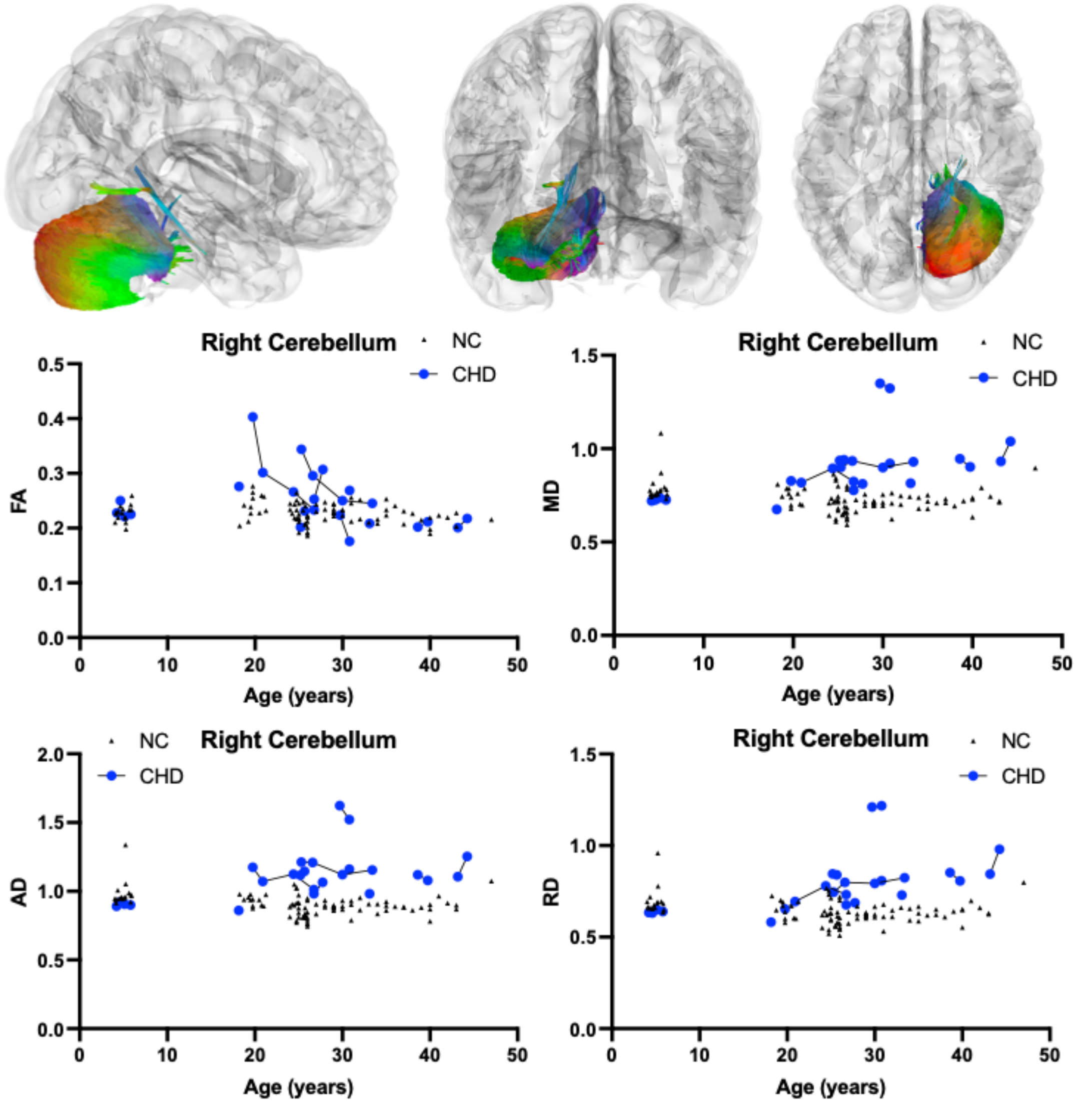

Figure D2. Atlas Based Fiber Tractography of the right cerebellum demonstrating age related effects on A.) fractional anisotropy (FA), B.) mean diffusivity (MD), C.) radial diffusivity (RD), D.) axial diffusivity (AD) between CHD participants (blue) and NC controls (black triangles). There was no statistically significant interaction between participant age and the presence of CHD on right cerebellar FA ($\chi^2(1) < 0.01$, p-value$_{corrected}$ = 1.000). There was a statistically significant positive interaction between participant age and the presence of CHD on right cerebellar MD ($\chi^2(1) = 18.59$, p-value$_{corrected}$ = 0.0006), AD ($\chi^2(1) = 13.31$, p-value$_{corrected}$ = 0.0095), and RD ($\chi^2(1) = 25.79$, p-value$_{corrected}$ < 0.0001).

medRxiv preprint doi: https://doi.org/10.1101/2025.08.06.25332949; this version posted August 10, 2025. The copyright holder for this preprint (which was not certified by peer review) is the author/funder, who has granted medRxiv a license to display the preprint in perpetuity.
This article is a US Government work. It is not subject to copyright under 17 USC 105 and is also made available for use under a CC0 license.

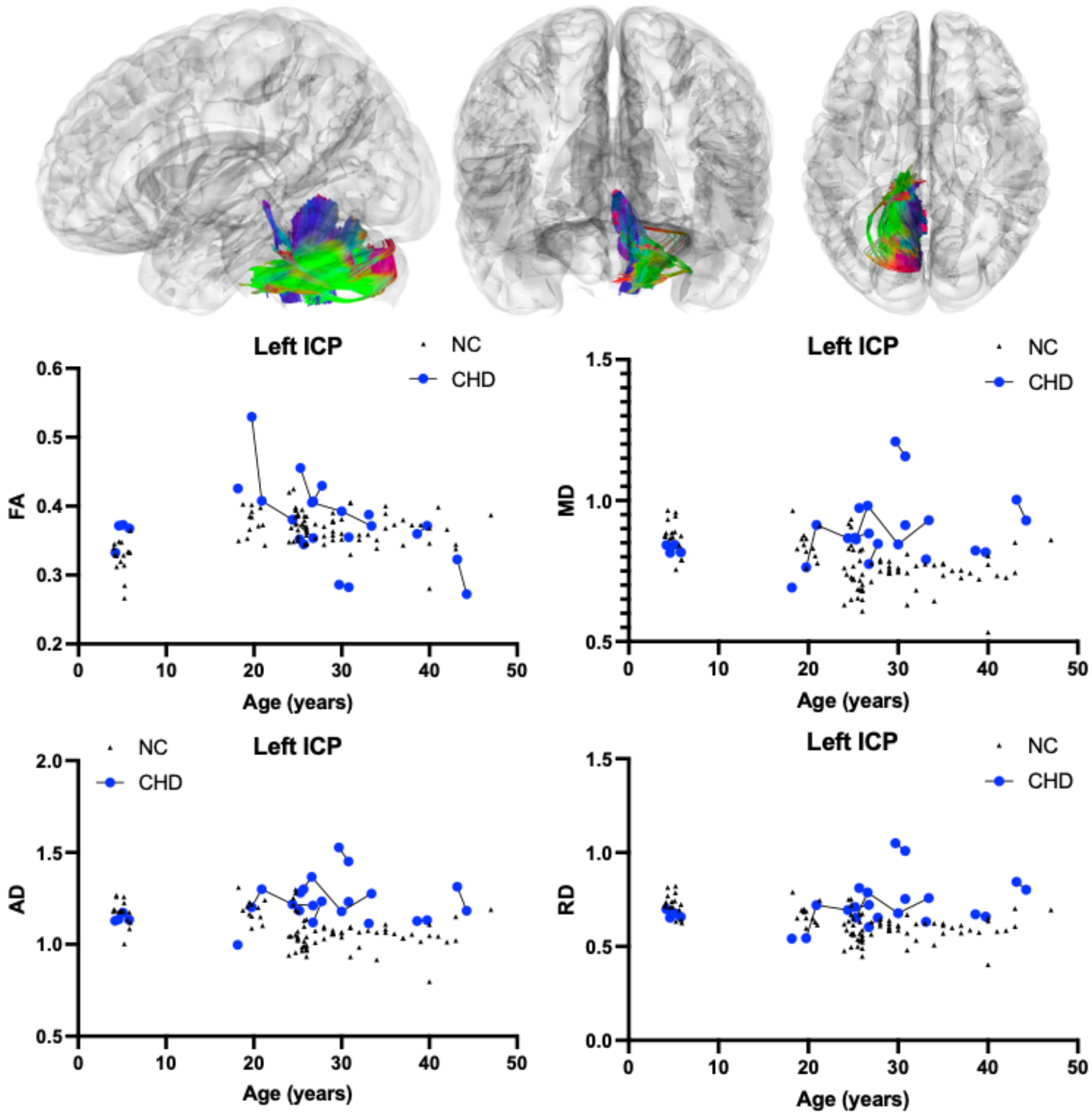

Figure D3. Atlas Based Fiber Tractography of the left inferior cerebellar peduncle (ICP) demonstrating age related effects on A.) fractional anisotropy (FA), B.) mean diffusivity (MD), C.) radial diffusivity (RD), D.) axial diffusivity (AD) between CHD participants (blue) and NC controls (black triangles). There was no statistically significant interaction between participant age and the presence of CHD on left ICP FA ($\chi^2(1) = 0.79$, p-value$_{corrected}$ = 1.000) or AD ($\chi^2(1) = 6.672$, p-value$_{corrected}$ = 0.3537). There was a statistically significant positive interaction between participant age and the presence of CHD on left ICP MD ($\chi^2(1) = 13.06$, p-value$_{corrected}$ = 0.0109) and RD ($\chi^2(1) = 16.84$, p-value$_{corrected}$ = 0.0015).

medRxiv preprint doi: https://doi.org/10.1101/2025.08.06.25332949; this version posted August 10, 2025. The copyright holder for this preprint (which was not certified by peer review) is the author/funder, who has granted medRxiv a license to display the preprint in perpetuity. This article is a US Government work. It is not subject to copyright under 17 USC 105 and is also made available for use under a CC0 license.

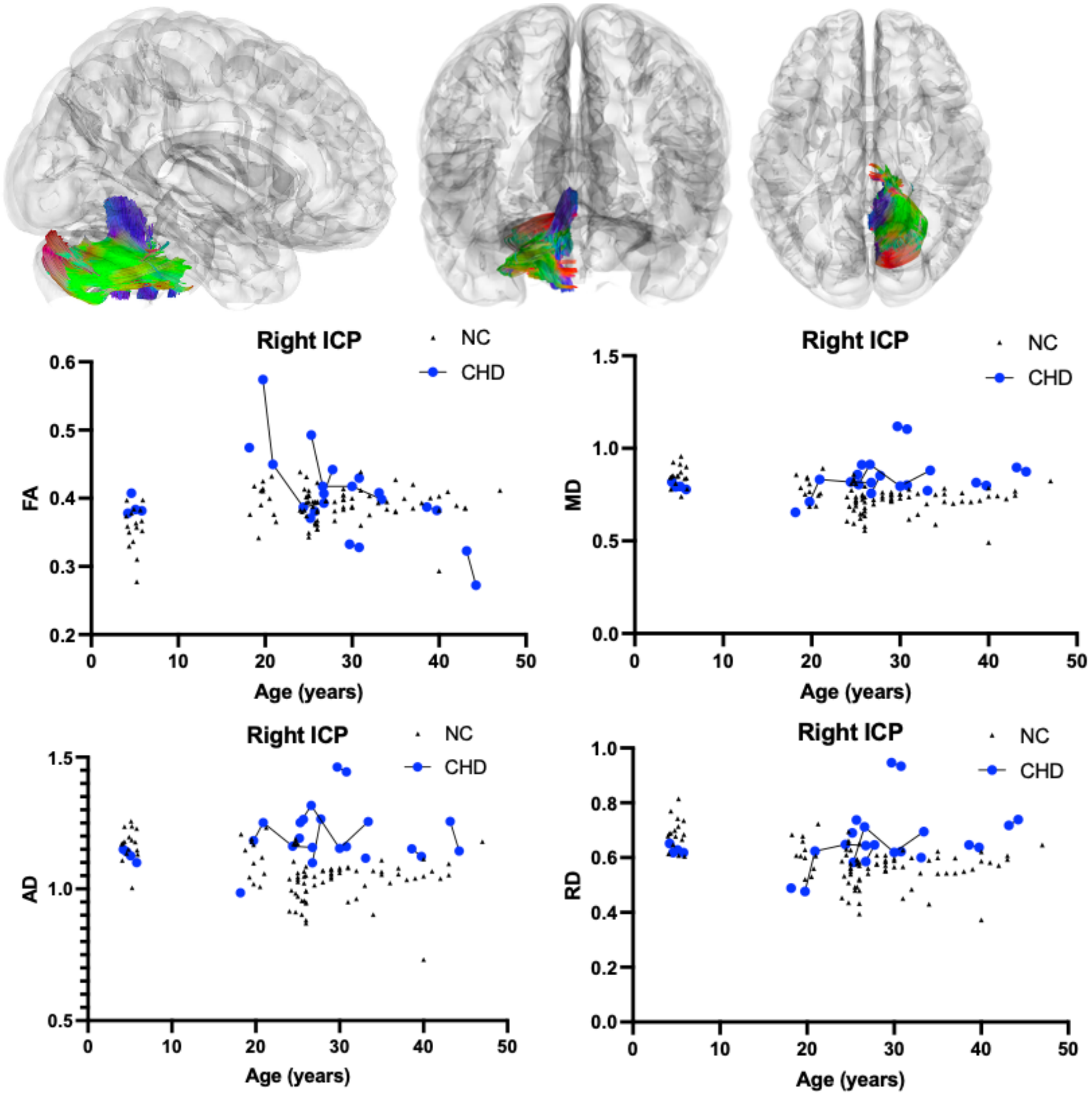

Figure D4. Atlas Based Fiber Tractography of the right inferior cerebellar peduncle (ICP) demonstrating age related effects on A.) fractional anisotropy (FA), B.) mean diffusivity (MD), C.) radial diffusivity (RD), D.) axial diffusivity (AD) between CHD participants (blue) and NC controls (black triangles). There was no statistically significant interaction between participant age and the presence of CHD on right ICP FA ($\chi^2(1) = 0.30$, p-value$_{corrected} = 1.000$) or AD ($\chi^2(1) = 5.723$, p-value$_{corrected} = 0.6030$). There was a statistically significant positive interaction between participant age and the presence of CHD on right ICP MD ($\chi^2(1) = 12.39$, p-value$_{corrected} = 0.0155$) and RD ($\chi^2(1) = 15.65$, p-value$_{corrected} = 0.0027$).

medRxiv preprint doi: https://doi.org/10.1101/2025.08.06.25332949; this version posted August 10, 2025. The copyright holder for this preprint (which was not certified by peer review) is the author/funder, who has granted medRxiv a license to display the preprint in perpetuity.
This article is a US Government work. It is not subject to copyright under 17 USC 105 and is also made available for use under a CC0 license.

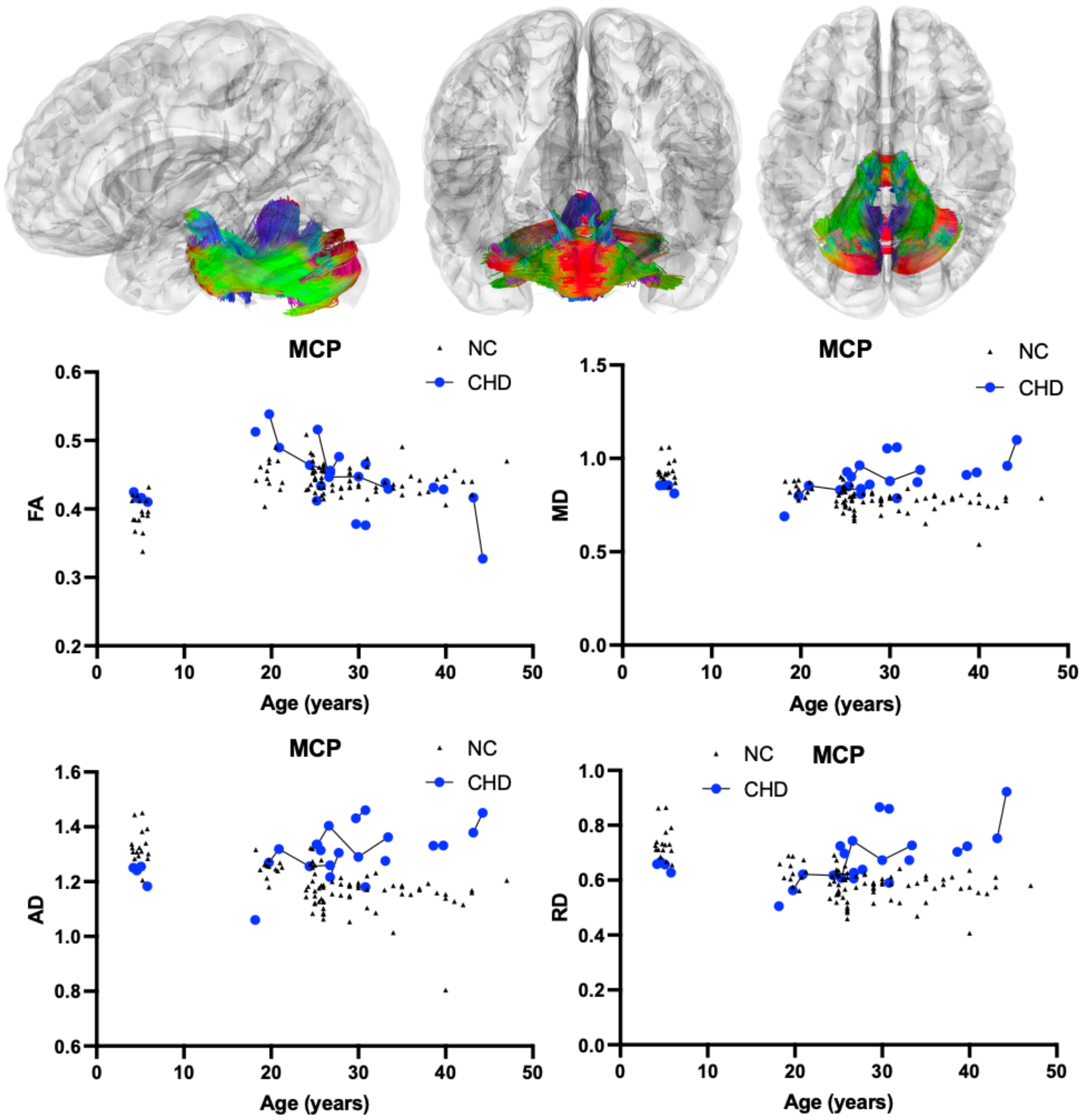

Figure D5. Atlas Based Fiber Tractography of the middle cerebellar peduncle (MCP) demonstrating age related effects on A.) fractional anisotropy (FA), B.) mean diffusivity (MD), C.) radial diffusivity (RD), D.) axial diffusivity (AD) between CHD participants (blue) and NC controls (black triangles). There was no statistically significant interaction between participant age and the presence of CHD on MCP FA ($\chi^2(1) = 0.31$, p-value$_{corrected}$ = 1.000). There was a statistically significant positive interaction between participant age and the presence of CHD on MCP MD ($\chi^2(1) = 20.23$, p-value$_{corrected}$ = 0.0002), AD ($\chi^2(1) = 11.66$, p-value$_{corrected}$ = 0.0230), and RD ($\chi^2(1) = 24.89$, p-value$_{corrected}$ < 0.0001).

medRxiv preprint doi: https://doi.org/10.1101/2025.08.06.25332949; this version posted August 10, 2025. The copyright holder for this preprint (which was not certified by peer review) is the author/funder, who has granted medRxiv a license to display the preprint in perpetuity. This article is a US Government work. It is not subject to copyright under 17 USC 105 and is also made available for use under a CC0 license.

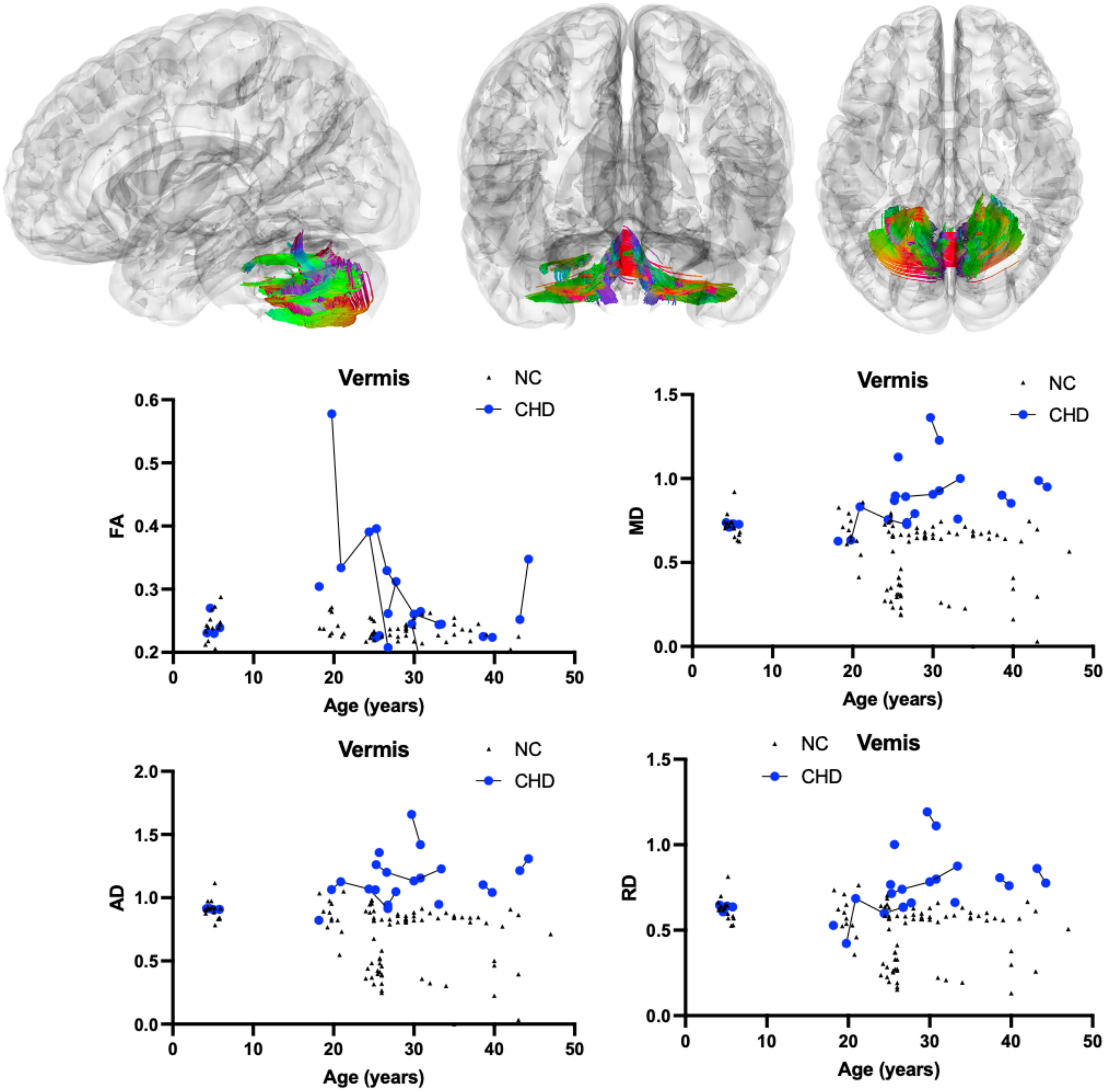

Figure D6. Atlas Based Fiber Tractography of the vermis demonstrating age related effects on A.) fractional anisotropy (FA), B.) mean diffusivity (MD), C.) radial diffusivity (RD), D.) axial diffusivity (AD) between CHD participants (blue) and NC controls (black triangles). There was no statistically significant interaction between participant age and the presence of CHD on vermis FA ($\chi^2(1) = 1.46$, $p\text{-value}_{corrected} = 1.000$) or AD ($\chi^2(1) = 8.783$, $p\text{-value}_{corrected} = 0.109$). There was a statistically significant positive interaction between participant age and the presence of CHD on vermis MD ($\chi^2(1) = 13.16$, $p\text{-value}_{corrected} = 0.0103$) and RD ($\chi^2(1) = 14.02$, $p\text{-value}_{corrected} = 0.0072$).

medRxiv preprint doi: https://doi.org/10.1101/2025.08.06.25332949; this version posted August 10, 2025. The copyright holder for this preprint
(which was not certified by peer review) is the author/funder, who has granted medRxiv a license to display the preprint in perpetuity.
This article is a US Government work. It is not subject to copyright under 17 USC 105 and is also made available
for use under a CC0 license.

Supplement E: DTI LMEM Estimates, Standard Errors, and Graphs

Supplement Table E1. Estimates and Standard Errors from the LMEM for comparisons of FA between CHD and NC for each of the structures analyzed. Estimates are given followed by the standard errors in parenthesis.

Structure	*Intercept*	*Age*Cohort (Interaction)*	*Cohort*	*Age*	*Sex*
Whole Brain	0.4070 (0.0062)	-0.0013 (0.0005)	0.0026 (0.0139)	0.0094 (0.0002)	-0.0005 (0.0025)
Left Cerebellum	0.2456 (0.0102)	-0.0006 (0.0008)	0.0346 (0.0229)	-0.0004 (0.0004)	-0.0031 (0.0042)
Right Cerebellum	0.2463 (0.0107)	0.00001 (0.0009)	0.0236 (0.0238)	-0.0010 (0.0003)	-0.0013 (0.0043)
Left Inferior Cerebellar Peduncle	0.3608 (0.0159)	-0.0012 (0.0013)	0.0427 (0.0358)	-0.0002 (0.0006)	-0.0036 (0.0065)
Right Inferior Cerebellar Peduncle	0.3877 (0.0174)	-0.0008 (0.0014)	0.0439 (0.0389)	-0.0004 (0.0006)	-0.0035 (0.0071)
Middle Cerebellar Peduncle	0.4340 (0.0188)	-0.0009 (0.0015)	0.0328 (0.0417)	-0.0004 (0.0007)	-0.0024 (0.0076)
Superior Cerebellar Peduncle	0.4240 (0.0133)	-0.0001 (0.0011)	0.0096 (0.0299)	0.0003 (0.0005)	-0.0009 (0.0054)
Vermis	0.2492 (0.0175)	0.0017 (0.0014)	0.0407 (0.0387)	-0.0020 (0.0006)	-0.0062 (0.0071)
Corpus Callosum	0.4834 (0.0062))	-0.0014 (0.0005)	-0.009 (0.014)	0.0011 (0.0002)	0.0009 (0.0025)

medRxiv preprint doi: https://doi.org/10.1101/2025.08.06.25332949; this version posted August 10, 2025. The copyright holder for this preprint (which was not certified by peer review) is the author/funder, who has granted medRxiv a license to display the preprint in perpetuity. This article is a US Government work. It is not subject to copyright under 17 USC 105 and is also made available for use under a CC0 license.

Supplement Table E2. Estimates and Standard Errors from the LMEM for comparisons of MD between CHD and NC for each of the structures analyzed. Estimates are given followed by the standard errors in parenthesis.

Structure	*Intercept*	*Age*Cohort (Interaction)*	*Cohort*	*Age*	*Sex*
Whole Brain	0.8988 (0.0092)	0.0065 (0.0008)	-0.0604 (0.0207)	-0.0029 (0.0003)	-0.0017 (0.0038)
Left Cerebellum	0.8333 (0.0294)	0.0113 (0.0024)	-0.1201 (0.0641)	-0.0041 (0.0011)	-0.0007 (0.0120)
Right Cerebellum	0.8144 (0.0307)	0.0105 (0.0024)	-0.0959 (0.0653)	-0.0040 (0.0011)	-0.0033 (0.0125)
Left Inferior Cerebellar Peduncle	0.9208 (0.0316)	0.0094 (0.0026)	-0.1181 (0.0699)	-0.0062 (0.0012)	-0.0058 (0.0129)
Right Inferior Cerebellar Peduncle	0.8843 (0.0315)	0.0091 (0.0026)	-0.1221 (0.0691)	-0.0061 (0.0012)	-0.0043 (0.0129)
Middle Cerebellar Peduncle	0.9794 (0.0306)	0.0114 (0.0025)	-0.1879 (0.0668)	-0.0076 (0.0011)	-0.0036 (0.0125)
Superior Cerebellar Peduncle	1.1237 (0.0260)	0.0101 (0.0021)	-0.1375 (0.0582)	-0.0044 (0.0010)	0.0067 (0.0106)
Vermis	0.7539 (0.0487)	0.0141 (0.0039)	-0.0598 (0.1046)	-0.0067 (0.0018)	-0.0199 (0.0199)
Corpus Callosum	0.9432 (0.0105)	0.0056 (0.0009)	-0.0551 (0.0235)	-0.0026 (0.0004)	-0.0020 (0.0043)

medRxiv preprint doi: https://doi.org/10.1101/2025.08.06.25332949; this version posted August 10, 2025. The copyright holder for this preprint (which was not certified by peer review) is the author/funder, who has granted medRxiv a license to display the preprint in perpetuity.
This article is a US Government work. It is not subject to copyright under 17 USC 105 and is also made available for use under a CC0 license.

Supplement Table E3. Estimates and Standard Errors from the LMEM for comparisons of AD between CHD and NC for each of the structures analyzed. Estimates are given followed by the standard errors in parenthesis.

Structure	*Intercept*	*Age*Cohort (Interaction)*	*Cohort*	*Age*	*Sex*
Whole Brain	1.3088 (0.0106)	0.0070 (0.0009)	-0.0807 (0.0235)	-0.0030 (0.0004)	-0.0027 (0.0043)
Left Cerebellum	1.0432 (0.0351)	0.0119 (0.0029)	-0.1009 (0.0782)	-0.0049 (0.0013)	-0.0036 (0.0143)
Right Cerebellum	1.0173 (0.0370)	0.0118 (0.0031)	-0.0922 (0.0824)	-0.0056 (0.0151)	-0.0056 (0.0151)
Left Inferior Cerebellar Peduncle	1.2552 (0.0421)	0.0089 (0.0034)	-0.0868 (0.0926)	-0.0073 (0.0015)	-0.0091 (0.0172)
Right Inferior Cerebellar Peduncle	1.2282 (0.04321)	0.0084 (0.0035)	-0.0716 (0.0946)	-0.0073 (0.0016)	-0.0076 (0.0176)
Middle Cerebellar Peduncle	1.4103 (0.0445)	0.0114 (0.0033)	-0.1738 (0.0915)	-0.0094 (0.0016)	-0.0057 (0.0182)
Superior Cerebellar Peduncle	1.6108 (0.0369)	0.0132 (0.0031)	-0.1944 (0.0830)	-0.0051 (0.0014)	0.0082 (0.0150)
Vermis	0.9562 (0.0587)	0.0147 (0.0048)	-0.0053 (0.1293)	-0.0086 (0.0021)	-0.0233 (0.0239)
Corpus Callosum	1.4899 (0.0101)	0.0060 (0.0008)	-0.0970 (0.0223)	-0.0028 (0.0004)	-0.0008 (0.0041)

medRxiv preprint doi: https://doi.org/10.1101/2025.08.06.25332949; this version posted August 10, 2025. The copyright holder for this preprint (which was not certified by peer review) is the author/funder, who has granted medRxiv a license to display the preprint in perpetuity.
This article is a US Government work. It is not subject to copyright under 17 USC 105 and is also made available for use under a CC0 license.

Supplement Table E4. Estimates and Standard Errors from the LMEM for comparisons of RD between CHD and NC for each of the structures analyzed. Estimates are given followed by the standard errors in parenthesis.

Structure	*Intercept*	*Age*Cohort (Interaction)*	*Cohort*	*Age*	*Sex*
Whole Brain	0.6937 (0.0097)	0.0064 (0.0008)	-0.0514 (0.0219)	-0.0029 (0.0004)	-0.0013 (0.0040)
Left Cerebellum	0.7283 (0.0267)	0.0117 (0.0021)	-0.1403 (0.0580)	-0.0036 (0.0010)	< 0.0001 (0.0109)
Right Cerebellum	0.7136 (0.0277)	0.0115 (0.0021)	-0.1319 (0.0585)	-0.0034 (0.0010)	-0.0031 (0.0113)
Left Inferior Cerebellar Peduncle	0.7528 (0.0271)	0.0094 (0.0022)	-0.1261 (0.0604)	-0.0056 (0.0010)	-0.0040 (0.0110)
Right Inferior Cerebellar Peduncle	0.7121 (0.0265)	0.0089 (0.0022)	-0.1334 (0.0591)	-0.0055 (0.0010)	-0.0027 (0.0108)
Middle Cerebellar Peduncle	0.7643 (0.0247)	0.0105 (0.0020)	-0.1753 (0.0552)	-0.0067 (0.0009)	-0.0026 (0.0101)
Superior Cerebellar Peduncle	0.8789 (0.0222)	0.0086 (0.0018)	-0.1156 (0.0494)	-0.0040 (0.0008)	0.0057 (0.0090)
Vermis	0.6544 (0.0435)	0.0136 (0.0036)	-0.0895 (0.0958)	-0.0057 (0.0016)	-0.0179 (0.0178)
Corpus Callosum	0.6704 (0.0120)	0.0054 (0.0010)	-0.0325 (0.0270)	-0.0025 (0.0004)	-0.0027 (0.0049)

medRxiv preprint doi: https://doi.org/10.1101/2025.08.06.25332949; this version posted August 10, 2025. The copyright holder for this preprint (which was not certified by peer review) is the author/funder, who has granted medRxiv a license to display the preprint in perpetuity. This article is a US Government work. It is not subject to copyright under 17 USC 105 and is also made available for use under a CC0 license.

Supplement F: DTI LMEM Graphs

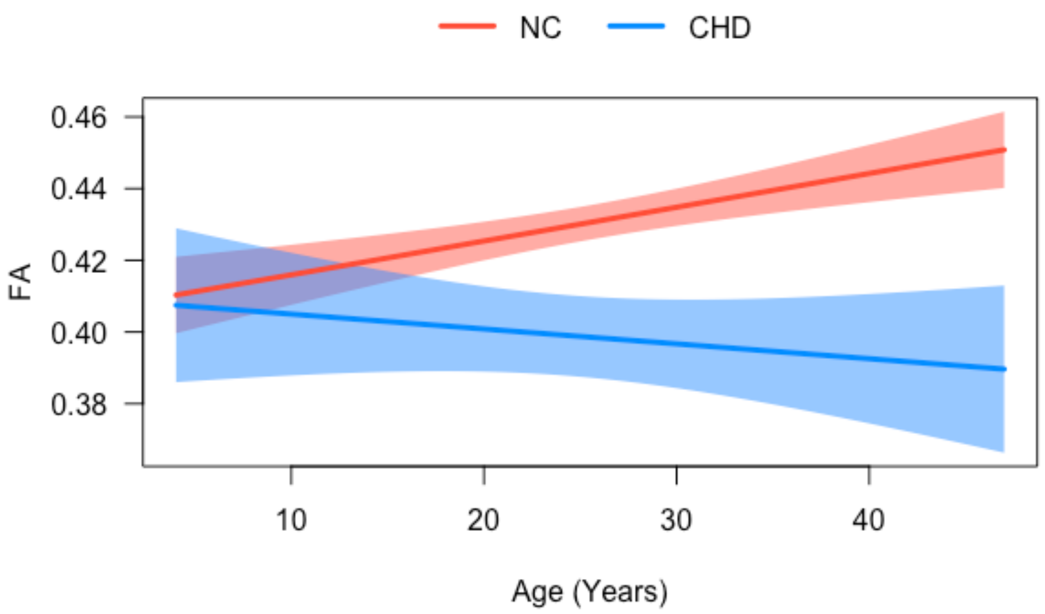

Figure F1. Whole Brain Differences in FA between CHD and NC.

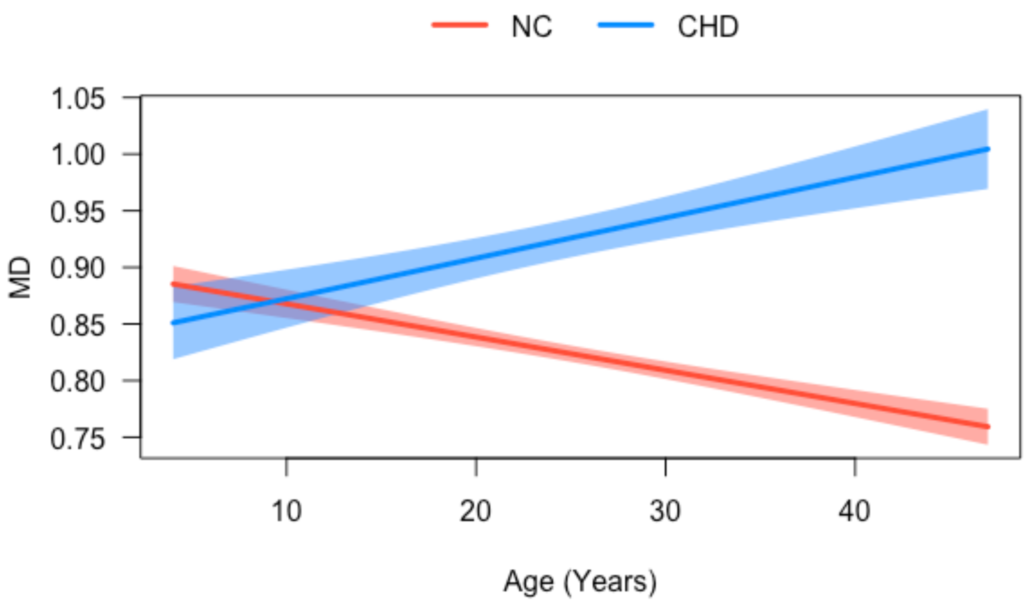

Figure F2. Whole Brain Differences in MD between CHD and NC.

medRxiv preprint doi: https://doi.org/10.1101/2025.08.06.25332949; this version posted August 10, 2025. The copyright holder for this preprint (which was not certified by peer review) is the author/funder, who has granted medRxiv a license to display the preprint in perpetuity.
This article is a US Government work. It is not subject to copyright under 17 USC 105 and is also made available for use under a CC0 license.

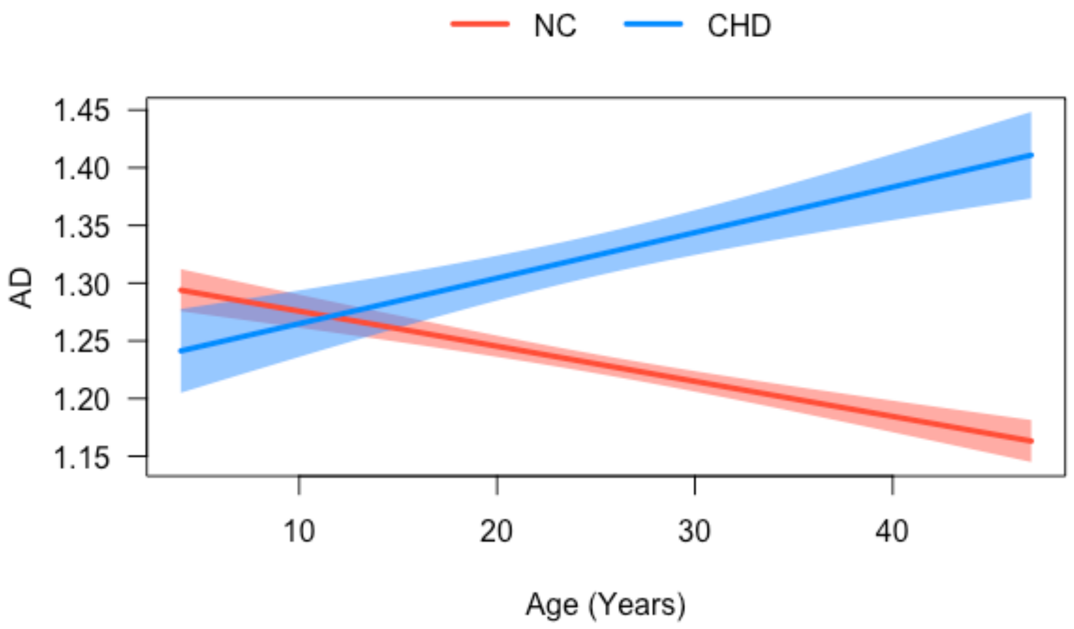

Figure F3. Whole Brain Differences in AD between CHD and NC.

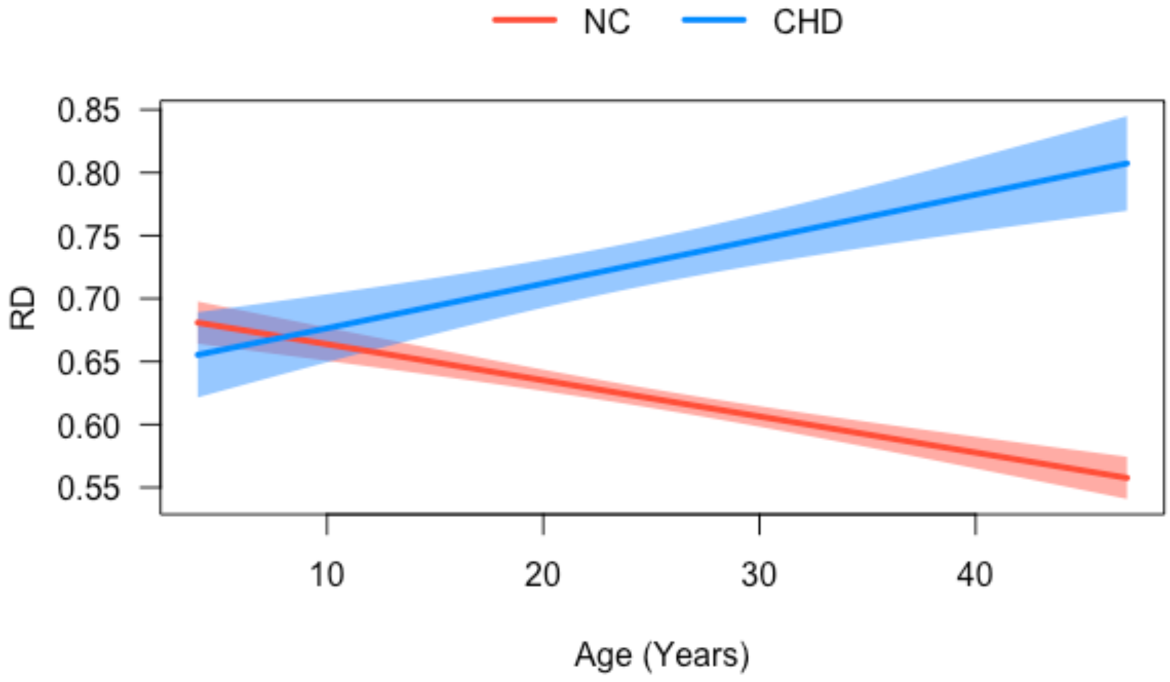

Figure F4. Whole Brain Differences in RD between CHD and NC.

medRxiv preprint doi: https://doi.org/10.1101/2025.08.06.25332949; this version posted August 10, 2025. The copyright holder for this preprint (which was not certified by peer review) is the author/funder, who has granted medRxiv a license to display the preprint in perpetuity.
This article is a US Government work. It is not subject to copyright under 17 USC 105 and is also made available for use under a CC0 license.

Supplement G: Correlational Fiber Tractography Supplementary Data

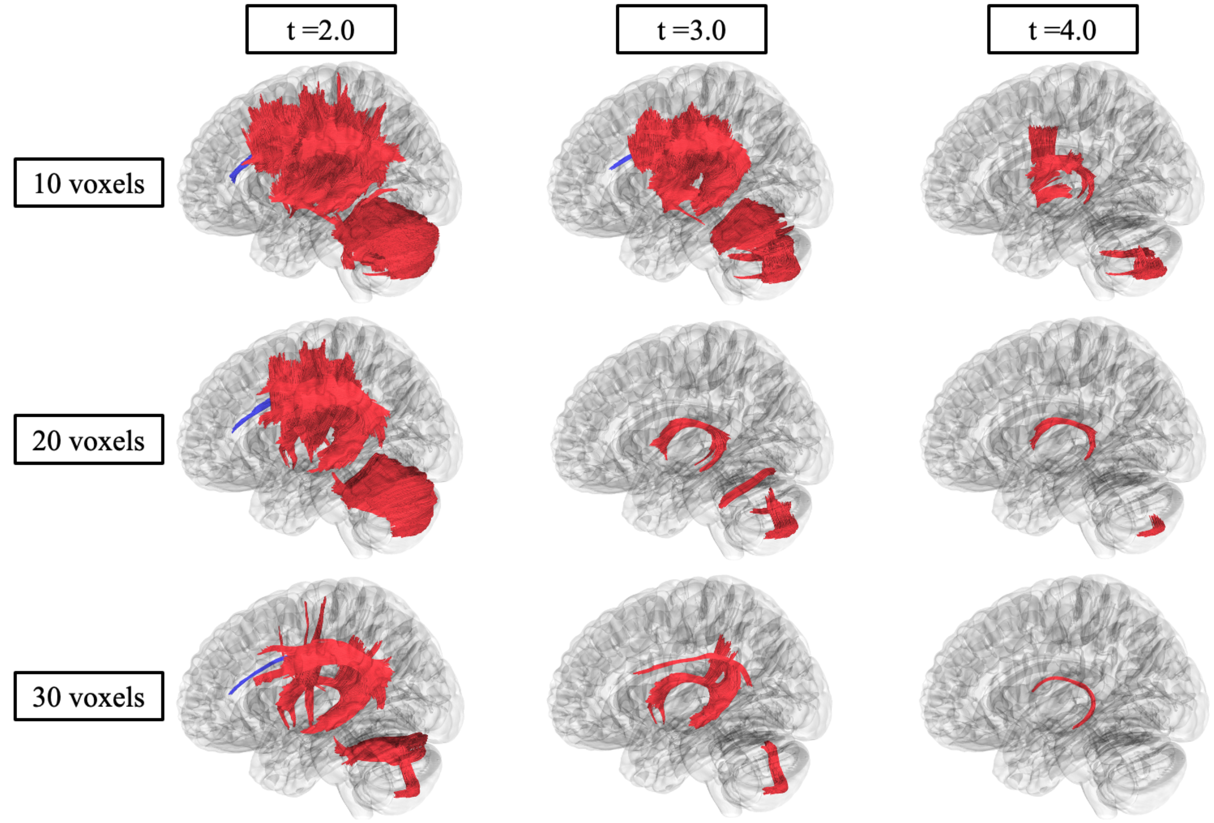

Figure G1. Correlational fiber tractography assessed differences in axial diffusivity (AD) in CHD participants and NC at varying length (voxels) and T threshold. Fiber tracts shown in red were evaluated to have a higher axial diffusivity in CHD participants compared to NC participants and were observed primarily in the cerebellar and corpus callosum pathways (FDR < 0.05). Sparse fiber tracts shown in blue were evaluated to have a higher AD in NC compared to CHD participants and were primarily found in association pathways.

medRxiv preprint doi: https://doi.org/10.1101/2025.08.06.25332949; this version posted August 10, 2025. The copyright holder for this preprint (which was not certified by peer review) is the author/funder, who has granted medRxiv a license to display the preprint in perpetuity.
This article is a US Government work. It is not subject to copyright under 17 USC 105 and is also made available for use under a CC0 license.

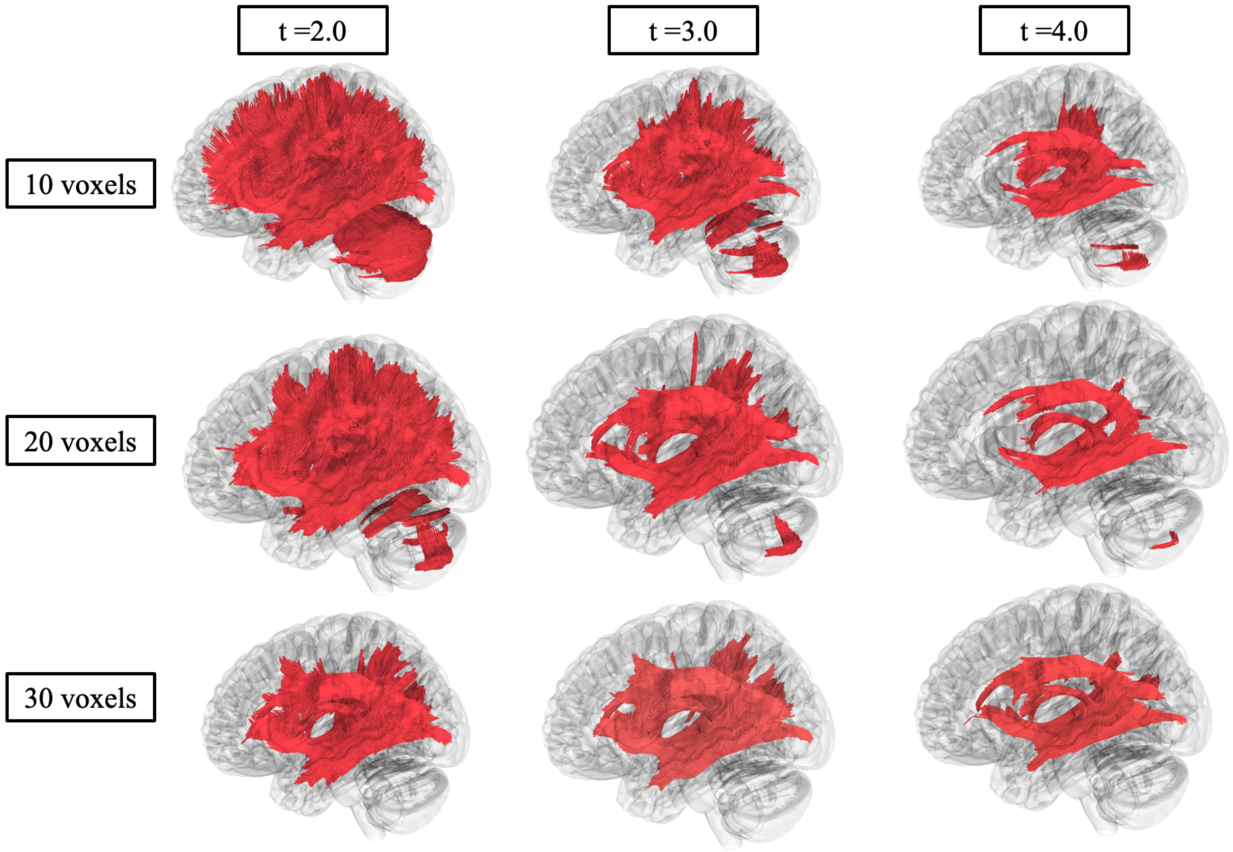

Figure G2. Correlational fiber tractography assessed differences in radial diffusivity (RD) in CHD participants and NC at varying length (voxels) and T threshold. Fiber tracts shown in red were evaluated to have a higher radial diffusivity in CHD participants compared to NC participants and were observed primarily in corpus callosum, cerebellar, and association pathways (FDR < 0.05). No fiber tracts were evaluated to have a higher RD in NC compared to CHD participants.

Supplement H: Correlational Fiber Tractography Localization

Table H1: Fiber Tract Localization for Correlational Fiber Tractography Analysis run with a *T*-score = 2.0 and a length threshold of 10 voxels.

	Higher in CHD	Higher in NC
**FA**	112845 Tracts<br>Association: 54.06%<br>Commissure: 32.15%<br>Basal Ganglia: 11.09%<br>Brainstem: 2.45%<br>Cranial Nerves: 0.14%<br>Cerebellum: 0.02%	4932 Tracts<br>Cerebellar: 96.43%<br>Brainstem: 3.57%
**MD**	409376 Tracts<br>Association: 33.18%<br>Commissure: 19.50%<br>Basal Ganglia: 17.28%<br>Brainstem: 15.68%<br>Cerebellar 14.01%<br>Cranial Nerves: 0.17%	0 Tracts
**AD**	134693 Tracts<br>Cerebellar: 48.29%<br>Basal Ganglia: 18.61%<br>Brainstem: 17.56%<br>Association: 7.65%<br>Commissure: 7.00%<br>Cranial Nerve: 0.16%	1663 Tracts<br>100% Association
**RD**	382315 Tracts<br>Association: 42.89%<br>Commissure: 24.42%<br>Basal Ganglia: 15.31%<br>Cerebellar: 8.81%<br>Brainstem: 8.55%<br>Cranial Nerve: 0.17%	0 Tracts

Table H2: Fiber Tract Localization for Correlational Fiber Tractography Analysis run with a *T*-score = 4.0 and a length threshold of 30 voxels.

	Higher in CHD	Higher in NC
**FA**	2916 Tracts<br>Association: 92.22%<br>Commissure: 5.08%<br>Basal Ganglia: 2.71%	0 Tracts
**MD**	31029 Tracts<br>Association: 92.79%<br>Commissure: 1.80%<br>Basal Ganglia: 5.29%<br>Brainstem 0.10%	0 Tracts
**AD**	87 Tracts<br>Basal Ganglia: 100%	0 Tracts
**RD**	30865 Tracts<br>Association: 82.90%<br>Basal Ganglia: 10.31%<br>Commissure: 6.77%<br>Brainstem: 0.01%	0 Tracts

medRxiv preprint doi: https://doi.org/10.1101/2025.08.06.25332949; this version posted August 10, 2025. The copyright holder for this preprint (which was not certified by peer review) is the author/funder, who has granted medRxiv a license to display the preprint in perpetuity. This article is a US Government work. It is not subject to copyright under 17 USC 105 and is also made available for use under a CC0 license.

Supplementary References

1. National Human Genome Research Institute. Investigations into Chediak-Higashi Syndrome and Related Disorders, ClinicalTrials.gov Identifier: NCT00005917. Updated March 17, 2025. Accessed April 11, 2025. https://clinicaltrials.gov/study/NCT00005917.
2. Reynolds JE, Long X, Paniukov D, Bagshawe M, Lebel C. Calgary Preschool magnetic resonance imaging (MRI) dataset. Data Brief. 2020 Jan 31;29:105224. doi: 10.1016/j.dib.2020.105224. PMID: 32071993; PMCID: PMC7016255
3. Reynolds, J., Long, X., Paniukov, D., Bagshawe, M., Dewey, D., & Lebel, C. (2023, June 13). Calgary Preschool MRI Dataset. https://doi.org/10.17605/OSF.IO/AXZ5R.
4. Nugent, A. C., Thomas, A. G., Mahoney, M., Gibbons, A., Smith, J. T., Charles, A. J., Shaw, J. S., Stout, J. D., Namyst, A. M., Basavaraj, A., Earl, E., Riddle, T., Snow, J., Japee, S., Pavletic, A. J., Sinclair, S., Roopchansingh, V., Bandettini, P. A., & Chung, J. (2022). The NIMH intramural healthy volunteer dataset: A comprehensive MEG, MRI, and behavioral resource. Scientific Data, 9, Article 518. https://doi.org/10.1038/s41597-022-01623-9.
5. Allison C. Nugent and Adam G Thomas and Margaret Mahoney and Alison Gibbons and Jarrod Smith and Antoinette Charles and Jacob S Shaw and Jeffrey D Stout and Anna M Namyst and Arshitha Basavaraj and Eric Earl and Travis Riddle and Joseph Snow and Shruti Japee and Adriana Pavletic and Stephen Sinclair and Vinai Roopchansingh and Peter A Bandettini and Joyce Chung (2023). The NIMH Healthy Research Volunteer Dataset. OpenNeuro. [Dataset] doi: doi:10.18112/openneuro.ds004215.v1.0.3.
6. Poldrack, R., Congdon, E., Triplett, W. et al. A phenome-wide examination of neural and cognitive function. Sci Data 3, 160110 (2016). https://doi.org/10.1038/sdata.2016.110.
7. Bilder, R and Poldrack, R and Cannon, T and London, E and Freimer, N and Congdon, E and Karlsgodt, K and Sabb, F (2018). UCLA Consortium for Neuropsychiatric Phenomics LA5c Study. OpenNeuro. [Dataset] doi: https://openneuro.org/datasets/ds000030/versions/00016.
8. Snoek L, van der Miesen MM, Beemsterboer T, van der Leij A, Eigenhuis A, Steven Scholte H. The Amsterdam Open MRI Collection, a set of multimodal MRI datasets for individual difference analyses. Sci Data. 2021;8(1):85. Published 2021 Mar 19. doi:10.1038/s41597-021-00870-6.
9. Lukas Snoek and Maite van der Miesen and Andries van der Leij and Tinka Beemsterboer and Annemarie Eigenhuis and Steven Scholte (2020). AOMIC-PIOP1. OpenNeuro. [Dataset] doi: 10.18112/openneuro.ds002785.v2.0.0.
10. Lukas Snoek and Maite van der Miesen and Andries van der Leij and Tinka Beemsterboer and Annemarie Eigenhuis and Steven Scholte (2021). AOMIC-ID1000. OpenNeuro. [Dataset] doi: 10.18112/openneuro.ds003097.v1.2.1.
